# Abstracts from Foreign Journals

**Published:** 1898-10

**Authors:** 


### ABSTRACTS FROM FOREIGN JOURNALS.

### GERMAN.

Some Statistics Relative to the Prevalence of Tuberculosis. (Abstracted for the Journal by Dr. James B. Paige, D.V.S.) The following statistics with reference to the occurrence of tuberculosis among meat-producing animals in Saxony, Germany, were collected by Dr. Edelnaun at the municipal slaughter-houses. They apply to animals from twenty-nine States. The statistics appear in the *Bericht über das Veterinärwesen im Königreich Sachsen für das Jahr*, 1896.

An abstract of the report by Professor Eugene Fröhner is published in the *Monatshefte für Praktische Thierheilkunde*, Band ix., Heft 4.

1. Of 85,000 cattle slaughtered, 23,000 were found tuberculous; 26.7 per cent. The cases were found distributed among the different classes of animals as follows: Of 39,000 cows and calves slaughtered, 13,000 (32 per cent.) were tuberculous; of 26,000 oxen, 6600 (25.5 per cent.); of 20,000 bulls, 3770 (18.5 per cent., were found affected with the disease.
2. Of 216,000 calves killed, 458 (0.21 per cent.) had tuberculosis.
3. Of 130,000 sheep, 94 (or 0.07 per cent.) were found affected.
4. Among 3250 goats killed, 10 cases of the disease were found (0.3 per cent.)
5. Among 220,000 swine, 2.74 per cent. were found tuberculous, 11,500 cases.
6. 0.34 per cent. of the horses slaughtered were tuberculous; 3500 killed; 12 cases of tuberculosis.
7. Of 400 dogs examined, 1 was found tuberculous; 0.25 per cent.

The total number of cattle in Saxony is 657,000.

Tuberculin has not come into very general use among practitioners. Only 3344 doses were distributed from the Dresden Veterinary College among the veterinarians during the year 1896.

Professor Fröhner calls particular attention to the following tables to be found on pages 177 and 178 of the report:

TABLE I.—OCCURRENCE OF TUBERCULOSIS AND USABILITY OF TUBERCULOUS ANIMALS.

| Species of animals. | Number of animals slaughtered. | Number of tuberculous animals. | | Numb'r of tuberculous carcasses condemn'd | Parts from tuberculous animals placed on sale in freibank.[1] | | | Numb'r of salable from the tuberculous anim'ls |
|---|---|---|---|---|---|---|---|---|
| | | Number. | Per cent. of slaughtered. | | Number of pieces sold in uncook'd state. | Number sold after previous sterilizing and melting out of fat. | Numb'r from which tried fat was sold. | |
| Cattle—total | 85,016 | 22,723 | 26. 72 | 473 | 674 | 617 | ...... | 20,959 |
| Oxen | 26,042 | 6,656 | 25. 55 | 72 | 69 | 171 | ...... | 6,344 |
| Cows and calves | 38,688 | 12,293 | 31. 77 | 352 | 557 | 362 | ...... | 11,022 |
| Bulls | 20,868 | 3,774 | 18. 60 | 49 | 48 | 84 | ...... | 3,598 |
| Calves | 215,894 | 458 | 0. 21 | 134 | 88 | 59 | ...... | 177 |
| Sheep | 132,810 | 94 | 0. 07 | 6 | 3 | 3 | ...... | 82 |
| Goats and kids | 3,294 | 10 | 0. 30 | 4 | .... | ..... | ...... | 6 |
| Swine | 419,188 | 11,487 | 2. 74 | 194 | 613 | 1701 | 675 | 8,304 |
| Horses | 3,457 | 12 | 0. 34 | 2 | .... | ..... | ...... | 10 |
| Dogs | 397 | 1 | 2. 22 | ...... | ..... | ..... | ...... | 1 |
| Total | 860,011 | 34,785 | ......... | 813 | 1378 | 2380 | 675 | 29,539 |

TABLE II.—DISTRIBUTION OF TUBERCULOSIS AMONG MEAT-PRODUCING ANIMALS.

| Species of animal. | The tuberculosis was found as | | | | | | | | | | | | |
|---|---|---|---|---|---|---|---|---|---|---|---|---|---|
| | Local tuberculosis | | Well advanced and involving several organs. | | Generalized tuberculosis. | | | | | | | | |
| | | | | | | Involving | | | | | In both of the preceding cases were tubercles found in the following organs. | | |
| | Single organs or lymph-glands of organs. | Several organs. | With emaciation and alterations in the flesh. | Without emaciation or lesions in flesh. | Number of cases observed. | Flesh or the muscle lymph-glands. | Bones. | In acute form with fever and miliary tubercles. | With advanced emaciation. | Without much emaciation. | Spleen. | Kidneys. | Udder. |
| Beeves | 17224 | 2498 | 44 | 1763 | 1194 | 306 | 138 | 3 | 72 | 675 | 318 | 490 | 141 |
| Calves | 113 | 62 | ... | 20 | 263 | 102 | ... | 1 | 5 | 155 | 149 | 58 | 2 |
| Sheep | 64 | 18 | ... | ... | 12 | 5 | 1 | ... | ... | 6 | 6 | ... | ... |
| Goats | 6 | ... | 1 | ... | 3 | ... | ... | ... | 2 | 1 | 2 | 1 | ... |
| Swine | 2737 | 3643 | 3 | 1013 | 3091 | 689 | 536 | 3 | 6 | 1857 | 1818 | 333 | 159 |
| Horses | 7 | 2 | ... | 1 | 2 | 1 | ... | ... | ... | 1 | 1 | 1 | ... |
| Dogs | 1 | ... | ... | ... | ... | ... | ... | ... | ... | ... | ... | ... | ... |

[1] A freibank is a place under government control where meats from diseased animals are exposed for sale.

Double (Peroneal and Tibial) Neurectomy for Spavin. By Professor Dr. Fröhner. (Translated by John A. W. Dollar.) Attempts made some years ago to cure lameness resulting from spavin by neurectomy were finally given up as uncertain and useless. Recently, Bosi,[1] of the Veterinary School in Bologna, has again taken up the question of neurectomy in spavin, and published some notable experiments. Bosi recommends simultaneous division of the peroneal and tibial nerves, because his anatomical preparations led him to believe that both nerves are concerned in the innervation of the hock-joint. He has proved the value of his method by six successful cases.

I have tested his method in one case; the animal was sent to the clinique of this college on February 16th of the present year, on account of spavin lameness which had resisted firing. On the right hock was a large spavin, and the animal went very lame in the same leg. The spavin-test confirmed the diagnosis. On February 18th the animal was cast and the tibial nerve divided on the inner surface of the limb a hand's-breadth above the hock. The animal was then turned over, and the peroneal nerve divided in the depression between the extensor pedis and peroneus muscles, a portion being removed. Both wounds were carefully sutured, dressed with airal paste, and covered with an antiseptic dressing. On moving the horse after operation the lameness was seen to have disappeared. On February 20th the wound on the outer surface of the limb had healed by primary intention, but that on the inner was suppurating, for which reason the stitches were removed and the wound dressed antiseptically. The horse was tested at a walk and trot on March 5th, when the lameness had completely disappeared. Being again tested on March 12th, the animal went sound, and the lameness had permanently disappeared.

This case certainly appears favorable to Bosi's method; I shall therefore continue the testing of his operation on a larger number of horses lame from spavin, and later report the results.

I should like, however, at the same time to offer a few observations as to the comparative desirability of double neurectomy and puncture-firing. I do not believe that either procedure will entirely displace the other, but that they will mutually supplement each other. Puncture-firing is so extremely simple and effective a mode of treatment as to deserve preference before double neurectomy, and it altogether obviates the need for casting and turning

[1] Il Nuovo Ercolani, 1897, Nos. 21 and 22.

over the horse, etc. But firing does not cure every case, and where it fails neurectomy may be employed as a last resort. In other chronic lamenesses, like ringbone, navicular ringbone, navicular diseases, etc., neurectomy should only be employed after other methods have received a fair trial.—*The Veterinarian*, September, 1898.

